# Blocking the CGRP Receptor: Differences across Human Vascular Beds

**DOI:** 10.3390/ph16081075

**Published:** 2023-07-28

**Authors:** Tessa de Vries, Deirdre M. Boucherie, Antoon van den Bogaerdt, A. H. Jan Danser, Antoinette MaassenVanDenBrink

**Affiliations:** 1 Division of Vascular Medicine and Pharmacology, Department of Internal Medicine, Erasmus MC University Medical Center, P.O. Box 2040, 3000 CA Rotterdam, The Netherlands; t.devries.2@erasmusmc.nl (T.d.V.);; 2 ETB-BISLIFE, Heart Valve Department, 1940 Beverwijk, The Netherlands

**Keywords:** antagonism, CGRP, gepants, migraine, potency, Schild plot, vasodilation

## Abstract

Multiple drugs targeting the calcitonin gene-related peptide (CGRP) receptor have been developed for the treatment of migraine. Here, the effect of the small-molecule CGRP receptor antagonist zavegepant (0.1 nM–1 µM) on CGRP-induced relaxation in isolated human coronary arteries (HCAs) was investigated. A Schild plot was constructed and a pA_2_ value was calculated to determine the potency of zavegepant. The potency and Schild plot slopes of atogepant, olcegepant, rimegepant, telcagepant, ubrogepant and zavegepant in HCAs and human middle meningeal arteries (HMMAs), obtained from our earlier studies, were compared. Zavegepant shifted the concentration–response curve to CGRP in HCAs. The corresponding Schild plot slope was not different from unity, resulting in a pA_2_ value of 9.92 ± 0.24. No potency difference between HCAs and HMMAs was observed. Interestingly, olcegepant, atogepant and rimegepant, with a Schild plot slope < 1 in HCAs, were all >1 log unit more potent in HMMAs than in HCAs, while telcagepant, ubrogepant and zavegepant, with a Schild plot slope not different from unity, showed similar (<1 log difference) potency across both tissues. As a Schild plot slope < 1 may point to the involvement of multiple receptors, it is important to further identify the receptors involved in the relaxation to CGRP in HCAs, which may be used to improve the cardiovascular safety of future antimigraine drugs.

## 1. Introduction

Calcitonin gene-related peptide (CGRP) is a 37-amino acid neuropeptide and potent vasodilator. Under physiological circumstances, CGRP does not have a primary role in the regulation of blood pressure [1]. However, it can induce vasodilation during hypertension or ischemia, thereby increasing blood flow to organs [1,2]. Moreover, CGRP is involved in the pathophysiology of migraine. During a migraine attack, the trigeminovascular system is activated, resulting in the release of CGRP and subsequent activation of nociceptors, followed by the perception of pain [3,4]. CGRP levels were shown to increase during migraine headache, and infusion of CGRP in migraine patients induces migraine-like attacks [5,6,7,8].

CGRP exerts its effects by binding to the canonical CGRP receptor, consisting of a single-transmembrane protein called receptor activity modifying protein 1 (RAMP1) coupled to a seven-transmembrane G protein-coupled receptor called calcitonin receptor-like receptor (CLR) and intracellular receptor component protein (RCP). The CGRP receptor is part of a larger family of receptors consisting of combinations of similar subunits (i.e., calcitonin receptor [CTR], RAMP2, RAMP3) [9]. Due to the similarities in receptor composition of the receptors within this family, multiple receptors can be activated by the same ligand, albeit with different potencies. Therefore, CGRP can not only potently activate the canonical CGRP receptor (CLR/RAMP1) but also the AMY1 receptor (CTR/RAMP1), and may additionally exert effects at the other receptors within this family at high concentrations [9].

For the acute treatment and prevention of migraine, multiple drugs have been developed that antagonize the CGRP receptor. The drug class of small-molecule CGRP receptor antagonists is called gepants. Clinical trials showed that they were effective for the treatment of migraine [10,11,12,13]. However, the development of the first generation of gepants was halted. Olcegepant was designed as an intravenous formulation, which was suboptimal for the treatment of migraine in an outpatient setting. Subsequent gepants were designed for oral use (telcagepant, rimegepant, atogepant, ubrogepant) or as intranasal spray (zavegepant). For telcagepant, development was discontinued due to concerns about hepatotoxicity, since elevated aminotransferases were found in some of the patients [14]. Fortunately, the newer gepants did not show alterations in liver enzymes and were approved for clinical use for the acute and/or prophylactic treatment of migraine [15]. Head-to-head comparison trials with different gepants have not been performed, making it difficult to draw reliable conclusions about which gepant is the most effective. However, the efficacy and tolerability results from clinical trials show more or less similar outcomes for the different gepants [11,16].

Over the years, the potency of all aforementioned gepants was determined in different vascular tissues in preclinical studies performed in our lab [17,18,19,20,21,22,23]. The current study investigated the potency of the most recently developed gepant, zavegepant, in human coronary arteries, and the results are compared to previous results from other gepants in both human coronary arteries and human middle meningeal arteries.

## 2. Results

### 2.1. Potency of Zavegepant in Human Coronary Arteries

**Figure 1:**
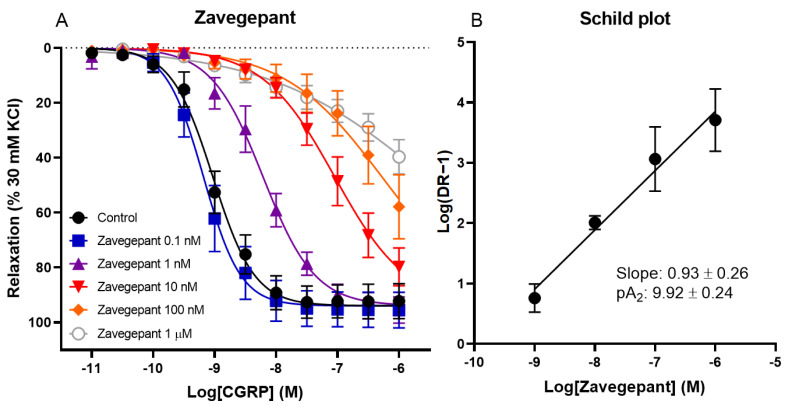
**The effect of different concentrations of zavegepant on CGRP-induced relaxation of isolated human coronary arteries.** (**A**) Concentration–response curve to CGRP in the presence or absence of 0.1 nM, 1 nM, 10 nM, 100 nM or 1 µM zavegepant. (**B**) The corresponding Schild plot. Data are expressed as mean ± SEM.

The effect of different concentrations of zavegepant on CGRP-induced relaxation of human coronary arteries was investigated. Vessel segments were incubated with or without 0.1 nM, 1 nM, 10 nM, 100 nM or 1 µM zavegepant. A concentration-dependent shift in the concentration–response curve to CGRP was observed (pEC_50_ control 9.01 ± 0.09, 0.1 nM 9.16 ± 0.14, 1 nM 8.18 ± 0.16, 10 nM 6.98 ± 0.13, 100 nM 5.96 ± 0.51 and 1 µM 5.31 ± 0.51; Figure 1). Moreover, the maximum relaxation to CGRP (E_max_) was not affected by zavegepant, although higher concentrations of CGRP are necessary to confirm this for concentrations of zavegepant above 10 nM, since a plateau of maximum relaxation was not reached for higher concentrations of zavegepant. The pEC_50_ values of the concentration–response curves were used to calculate a ratio for each concentration of zavegepant versus the control curve. Subsequently, a Schild plot was constructed. For the human coronary artery, the slope of the Schild plot was not different from unity (0.93 ± 0.26). Therefore, a pA_2_ value could be calculated as an estimate of the potency of zavegepant in this tissue (pA_2_: 9.92 ± 0.24).

### 2.2. Potency of All Gepants in Human Coronary Arteries and Human Middle Meningeal Arteries

**Figure 2:**
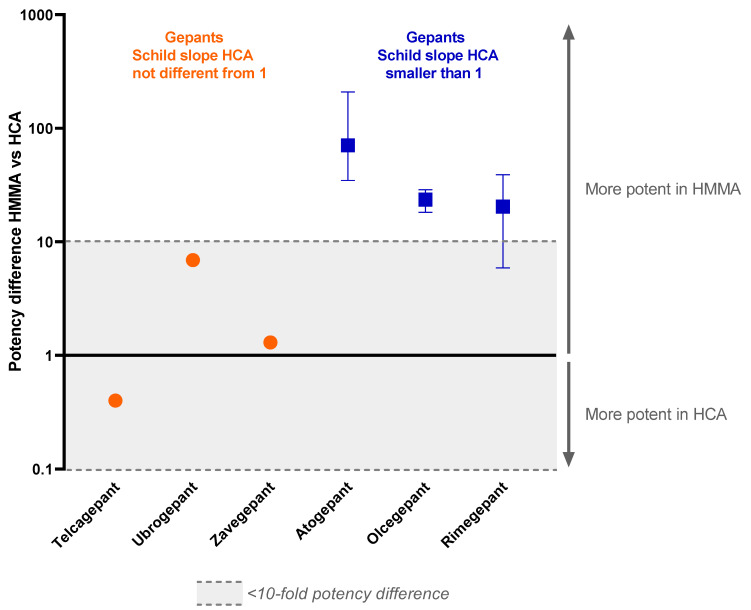
**Potency difference of gepants targeting the CGRP receptor in the human middle meningeal artery versus the human coronary artery.** All known pA_2_ values and pK_b_ values of drugs in the range of 10 nM to 1 µM are used in this graph. Data are expressed as median and range from min to max.

Comparable experiments using human coronary arteries and human middle meningeal arteries have been performed in our lab in the past using other drugs targeting the CGRP receptor, specifically, atogepant, olcegepant, rimegepant, telcagepant and ubrogepant. In this study, all previously obtained results are compared with our current findings on zavegepant and the potency of zavegepant in human middle meningeal arteries (Table 1, Figure 2).

Since the slope of the Schild plot can give information about the interaction between an antagonist and the receptor(s) it binds to, these data were extracted from previous studies. For some of the gepants, the Schild plot constructed from the data of the human coronary arteries had a slope less than unity. This was the case for atogepant, olcegepant and rimegepant [17,21,22]. For telcagepant, ubrogepant and zavegepant, the Schild plot slope did not differ from unity in human coronary arteries [19,21]. Interestingly, in contrast to the results from human coronary arteries, the Schild plot slope of the data obtained in human middle meningeal arteries for olcegepant and rimegepant did not differ from unity [18,22]. For atogepant, telcagepant, ubrogepant and zavegepant, not enough data are available to draw a conclusion on the slope of the Schild plot in human middle meningeal arteries. Remarkably, atogepant, olcegepant and rimegepant, with a Schild plot slope < 1 in the human coronary artery, were all >1 log unit more potent in human middle meningeal arteries than in human coronary arteries, with the difference becoming more apparent at higher concentrations of antagonist, while telcagepant, ubrogepant and zavegepant showed similar (<1 log difference) potency in both vascular beds.

## 3. Discussion

Zavegepant inhibited the concentration–response curve to CGRP in human coronary arteries in a concentration-dependent matter, as was observed previously for other gepants [17,19,21,22]. The corresponding Schild plot had a slope not different from unity, suggesting that increasing the dose by a certain factor results in an equally increased inhibition of CGRP-induced relaxation, consistent with simple competitive antagonism [24].

Subsequently, the potency of blocking the vasodilation response to CGRP in two different vascular beds was compared for different drugs that act as antagonists for the CGRP receptor, i.e., atogepant, olcegepant, rimegepant, telcagepant, ubrogepant and zavegepant. As we investigated basic pharmacological mechanisms in our study, we did not exclude gepants that have not reached the market for clinical use (olcegepant and telcagepant) from our analyses. For each of the CGRP receptor-inhibiting drugs, a Schild plot was constructed based on the data obtained for human coronary arteries and human middle meningeal arteries. A Schild plot can be used as a pharmacological tool to enhance comprehension of the interaction between a receptor antagonist and the receptor(s) it binds to. In human middle meningeal arteries, the slope of the Schild plot did not differ from unity for the gepants for which this was determined, i.e., olcegepant and rimegepant. When looking at the Schild plot slopes in human coronary arteries, the gepants can be divided into two subgroups. The first subgroup consists of telcagepant, ubrogepant and zavegepant, for which the Schild plot slope in human coronary arteries did not differ from unity. For the second subgroup, consisting of atogepant, olcegepant and rimegepant, the slope was significantly different from unity.

Multiple explanations for a slope different from unity exist. First, it could suggest a noncompetitive type of antagonism [24]. However, in the case of noncompetitive antagonism, a decrease in maximum response would be expected. In the experiments with the gepants with a slope less than 1, i.e., atogepant, olcegepant and rimegepant, a decrease in E_max_ was not observed [17,21,22], although it cannot be completely ruled out that the E_max_ decreases for the highest concentrations of gepant used, since maximum relaxation was not reached. According to the induced-fit model described by Vauquelin et al., initial reversible and unstable binding of an antagonist to a receptor could be succeeded by the formation of a more stable antagonist–receptor complex, resulting in (partial) insurmountable inhibition [25]. This theory suggests that the type of receptor antagonism can change from surmountable to partially or even fully insurmountable. A rightward shift induced by increasing concentrations of gepant combined with a decrease in E_max_ would fit with partially insurmountable antagonism. While theoretically, future experiments could further investigate whether the E_max_ is indeed affected by high concentrations of atogepant, olcegepant or rimegepant, it should be kept in mind that agonism induced by CGRP would most probably lose its selectivity at concentrations in the millimolar range. Moreover, this theory does not explain the differences between Schild plot slopes in human coronary arteries versus middle meningeal arteries for olcegepant and rimegepant. A second explanation for a Schild plot slope different from 1 could be a hemi-equilibrium of experimental conditions. Previously, it was shown that increasing the incubation time to two hours did not affect the slope of the Schild plot of olcegepant [17]. Moreover, in human middle meningeal arteries, the slope does not differ from unity for rimegepant or olcegepant, suggesting that equilibrium is reached in this vascular tissue after 30 min of incubation. A third explanation for a Schild plot slope different from unity is that the effect induced by the agonist involves multiple receptors that are not all blocked by the antagonist to the same extent. This could be a plausible hypothesis in human coronary arteries.

The CGRP receptor, consisting of transmembrane proteins CLR and RAMP1, is closely related to other receptors within the same family [9]. The adrenomedullin receptor consists of the same subunit CLR, but is coupled to RAMP2 instead of RAMP1, while the adrenomedullin 2 receptor is composed of CLR coupled to RAMP3. Other receptors in this same family consist of the CTR subunit, namely, the calcitonin receptor itself, the amylin 1 (AMY1) receptor, composed of CTR and RAMP1, the amylin 2 receptor (CTR and RAMP2), and the amylin 3 receptor (CTR and RAMP3). CGRP and amylin are equipotent at the AMY1 receptor, and adrenomedullin and adrenomedullin 2 can also act at the canonical CGRP receptor, demonstrating the cross-activation of the receptors within this family by different agonists [9]. Interestingly, rimegepant was shown to antagonize CGRP signaling at the AMY1 receptor only 17- to 30-fold less potently than at the CGRP receptor, suggesting that gepants can target receptors other than the CGRP receptor [26]. Moreover, telcagepant showed 40-fold selectivity for the CGRP receptor over the AMY1 receptor, while olcegepant is around 200-fold more selective [27,28]. These data show that in addition to agonists activating multiple receptors within this family, some CGRP receptor antagonists are able to target multiple receptors. Additional research should be performed to accurately characterize the potency of all gepants for the different receptors within the CGRP receptor family.

**Figure 3:**
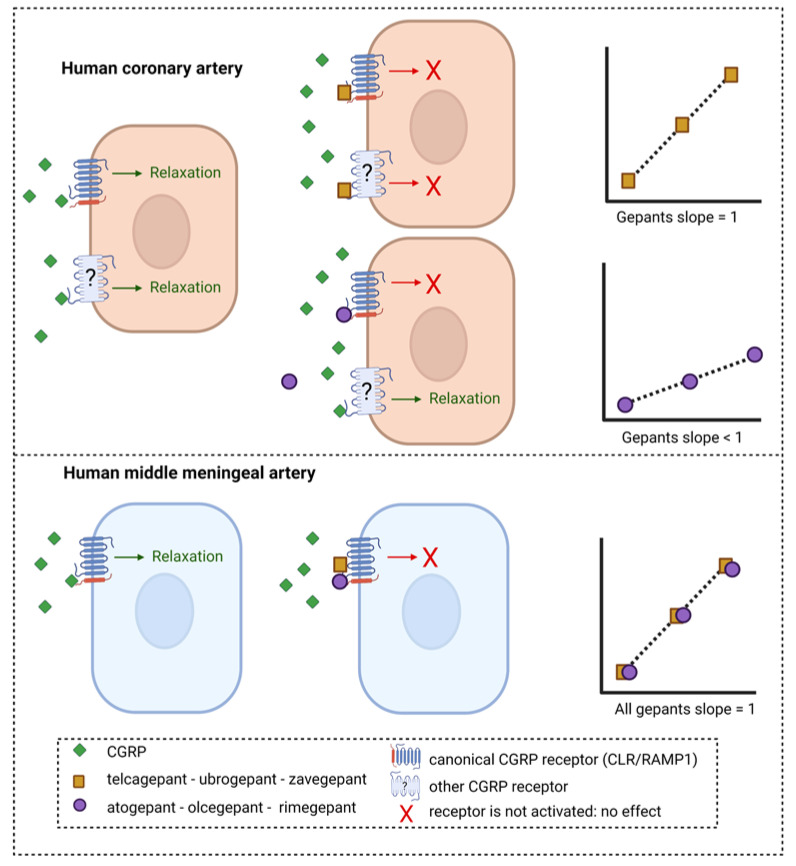
**Potential mechanism of inhibition of CGRP-induced relaxation in human arteries by different gepants.** Telcagepant, ubrogepant and zavegepant show a Schild plot slope in human coronary arteries that does not differ from unity, whereas for atogepant, olcegepant and rimegepant, the Schild slope is significantly less than unity. Possibly, the gepants with a slope < 1 are less potent in human coronary arteries than human middle meningeal arteries because CGRP can still induce relaxation via a second receptor that is not targeted by these gepants. If telcagepant, ubrogepant and zavegepant can target this second receptor in human coronary arteries with a potency similar to that on the canonical CGRP receptor, it could explain the absence of potency difference between the two tissues for these gepants. In human middle meningeal arteries, all gepants showed a slope not different from unity, suggesting that one receptor is involved in CGRP-induced relaxation and can be blocked by gepants.

The gepants atogepant, olcegepant and rimegepant all had a Schild plot slope below than 1 in human coronary arteries. For atogepant, not enough data were obtained in human middle meningeal arteries to construct a Schild plot, and therefore no conclusions can be drawn about the slope of the Schild plot of this drug in this tissue. However, for olcegepant and rimegepant, the Schild plot slope in human middle meningeal arteries did not differ from 1, in contrast to their responses in human coronary arteries. These findings suggest that these CGRP receptor-inhibiting drugs respond differently in the two vascular beds. If the Schild plot slope below than 1 in the human coronary arteries is indeed caused by the involvement of multiple receptors, these results would imply that in human coronary arteries, multiple receptors are involved in the relaxation to CGRP that are not antagonized by all gepants, while in the human middle meningeal arteries, one receptor acts as the main target for both CGRP and the gepants, resulting in a Schild plot slope not different from unity (Figure 3). Possibly, in the human coronary artery, CGRP could continue to induce relaxation via receptors other than the canonical CGRP receptor that are not blocked by gepants with a slope < 1, resulting in decreased potency of these gepants in this vascular tissue compared to human middle meningeal arteries. Following this line of reason, the three gepants with a slope not different from unity—telcagepant, ubrogepant and zavegepant—would be able to target these additional CGRP receptors, thereby minimizing the potency difference between human coronary arteries and human middle meningeal arteries. A possible candidate for the other receptor that could mediate the CGRP-induced relaxation in human coronary arteries could be the AMY1 receptor, consisting of CTR and RAMP1, considering the high potency of CGRP at this receptor, which is similar to the potency of CGRP at the canonical CGRP receptor [9], and the finding that at least some of the gepants seem to be able to target this receptor as well [26,27]. However, the fact that rimegepant has a relatively high affinity for the AMY1 receptor [26] argues against it being the second receptor in human coronary arteries. Further research should elucidate exactly what receptors are present in the different vascular beds.

The difference between the three gepants with a slope not different from unity and the three with a slope < 1 is unlikely to arise from a difference in the tissues itself, considering that all tissues were obtained from the same collaboration with the Heart Valve Department of ETB-BISLIFE. Moreover, no relation could be observed between the results and the order in which they were studied over the years, with olcegepant being the first gepant that was studied, followed by telcagepant, atogepant, ubrogepant, rimegepant, and zavegepant. Thus, the degree of receptor expression can be assumed to have remained unaltered over the years.

Since CGRP can serve as a rescue molecule during ischemia, CGRP receptor blockade could worsen the outcome if an ischemic event occurs, as observed previously in mice [22]. Therefore, in view of cardiovascular safety, a CGRP receptor antagonist should ideally potently block CGRP receptors in migraine-related structures to exert its antimigraine effects while effects on the coronary circulation remain limited. The meningeal vasculature is densely innervated by the trigeminal nerve, and the trigeminovascular system is activated during a migraine attack, resulting in the local release of CGRP [29]. Therefore, CGRP activity in meningeal arteries could possibly serve as a proxy for what is happening in the trigeminovascular system. Following this line of reasoning, a gepant with high potency in human middle meningeal arteries and low potency in human coronary arteries would be favorable. Telcagepant, ubrogepant and zavegepant show only minor potency differences between the two tissues. Atogepant displays the largest potency difference (35–210 times more potent in human middle meningeal artery), which seems favorable in view of the cardiovascular safety profile. However, efficacy, as well as safety, also depend on many other factors, such as plasma–protein binding, dosing, dosing intervals, duration of receptor antagonism in vivo and metabolism of the drug, as well as patient characteristics, such as the potential predominance of CGRP in the pathophysiology of their migraine attacks [30,31] and/or genetic polymorphisms in the CGRP receptor gene components [32].

## 4. Materials and Methods

### 4.1. Wire Myography Experiments in Human Coronary Arteries

Distal portions of the left anterior descending coronary artery were isolated from hearts of seven heart valve donors (four males and three females) aged 53 ± 3 years (mean ± SEM). Hearts were provided by ETB-BISLIFE (Heart Valve Department, Beverwijk, the Netherlands) following removal of the aortic and pulmonary valves for homograft valve transplantation. Hearts were obtained from Dutch postmortem donors who gave permission for research. Donor mediation took place via the Dutch Transplant Foundation (Leiden, Netherlands). Directly after circulatory arrest, the hearts were harvested and stored at 4 °C in a sterile organ-protecting solution. Coronary arteries were isolated and stored in oxygenated and carbonated Krebs solution (118 mM NaCl, 4.7 mM KCl, 2.5 mM CaCl_2_, 1.2 mM MgSO_4_, 1.2 mM KH_2_PO_4_, 25 mM NaHCO_3_ and 8.3 mM glucose, pH = 7.4) at 4 °C until the start of the experiment.

For functional experiments, 2 mm segments of human coronary arteries were mounted in Mulvany myograph organ baths (Danish Myo Technology, Aarhus, Denmark) using Ø 40 µm stainless-steel wires. The organ baths were kept at 37 °C and filled with oxygenated and carbonated Krebs solution. The mounted vessel segments were allowed to equilibrate before being stretched to a tension normalized to 0.9 times the estimated diameter at 100 mmHg transmural pressure [33]. The tension was recorded using LabChart data acquisition (AD instruments Ltd., Oxford, UK). Vessel segments were exposed to 30 mM KCl, washed, and exposed to 100 mM KCl, followed by additional washing steps. Next, vessel segments were incubated with zavegepant (0.1 nM, 1 nM, 10 nM, 100 nM or 1 µM) or control 30 min before a concentration–response curve to human αCGRP was constructed (0.01 nM–1 µM, half-logarithmic steps). The vessel segments were precontracted with 30 mM KCl 15 min before the start of the curve, and relaxation responses to CGRP were expressed as a percentage of precontraction.

For data analysis, sigmoidal curves were constructed using nonlinear regression analysis in Prism 8 (GraphPad Software, San Diego, CA, USA), from which a pEC_50_ value was obtained. A ratio was calculated between the pEC_50_ value of the different concentrations of zavegepant that shifted the concentration–response curve to CGRP and the pEC_50_ of the control curve, called the dose ratio (DR). The DR for each concentration of zavegepant was plotted in a Schild plot using log(DR-1). Linear regression was used to obtain the corresponding Schild plot slope, which gives information about the interaction between a receptor antagonist and the receptor(s) it binds to, and the pA_2_ value for zavegepant in human coronary arteries.

### 4.2. Comparison of Potency of Gepants in Human Coronary Arteries and Human Middle Meningeal Arteries

Results obtained with zavegepant in human coronary arteries were compared with a previous study investigating the potency of zavegepant in human middle meningeal arteries [23]. Moreover, the results from zavegepant were compared to previous experiments performed in the same lab in the Erasmus Medical Center, Rotterdam, the Netherlands between 2005 and 2023 using other CGRP receptor antagonists—atogepant [21], olcegepant [17,18], rimegepant [22], telcagepant [19,20] and ubrogepant [21]—in both human coronary arteries and human middle meningeal arteries. From previous studies, pK_b_ and pA_2_ values were obtained as a measure of potency in the different vascular tissues and the slope of the Schild plot was extracted. Gepants were sorted based on their Schild plot slope in human coronary arteries, and a potency difference between the two human vascular beds was calculated for each of the CGRP receptor antagonists. When multiple pK_b_ values of a single drug were known, a range for the potency difference between human middle meningeal arteries and human coronary arteries is presented. If the slope of the Schild plot does not differ from unity, the individual pK_b_ values for different antagonist concentrations should be equal to the pA_2_ [34].

## 5. Conclusions

For CGRP receptor antagonists with a Schild plot slope less than unity in human coronary arteries, the potency difference between coronary arteries and human middle meningeal arteries seems larger than for antagonists yielding a Schild plot slope equal to unity in human coronary arteries. As a Schild plot slope less than unity may point to the involvement of multiple receptors, it is important to identify the receptors involved in the relaxation to CGRP in human coronary arteries, which can be used to further improve the cardiovascular safety of future antimigraine drugs.

## Figures and Tables

**Table 1:** **Overview of the potency of different gepants targeting the CGRP receptor in human coronary arteries and human middle meningeal arteries and potency difference between the two vascular beds.** * As potency differences between tissues became apparent at higher antagonist concentrations and the determination of antagonist potency was more reliable at higher antagonist concentrations, due to smaller effects of variability in data on the calculated dose ratios, pK_b_ values obtained with 1 nM were not used for the comparison between human coronary arteries and human middle meningeal arteries. ** Calculated based on reported pEC_50_ values; results might be less accurate as the concentration–response curves did not reach a plateau. HCA: human coronary artery, HMMA: human middle meningeal artery.

	HCA	HMMA	Potency Difference
Olcegepant [17,18]	pK_b_ 1 nM *: 9.56 ± 0.22 pK_b_ 10 nM: 9.33 ± 0.25 pK_b_ 100 nM: 9.13 ± 0.17	pA_2_: 10.59 ± 0.54	10.7× more potent in HMMA 18.2× more potent in HMMA 28.8× more potent in HMMA
Telcagepant [19,20]	pA_2_: 8.43 ± 0.24	pK_b_ 1 µM: 8.03 ± 0.16	2.5× more potent in HCA
Atogepant [21]	pK_b_ 10 nM: 9.42 ± 0.22 pK_b_ 100 nM: 9.11 ± 0.34 pK_b_ 1 µM: 8.64 ± 0.21	pK_b_ 10 nM: 10.96 **	34.7× more potent in HMMA 70.8× more potent in HMMA 208.9× more potent in HMMA
Ubrogepant [21]	pA_2_: 8.86 ± 0.39	pK_b_ 10 nM: 9.70 **	6.9× more potent in HMMA
Rimegepant [22]	pK_b_ 1 nM *: 9.74 ± 0.05 pK_b_ 10 nM: 9.25 ± 0.15 pK_b_ 100 nM: 8.71 ± 0.16 pK_b_ 1 µM: 8.43 ± 0.25	pA_2_: 10.02 ± 0.33	1.9× more potent in HMMA 5.9× more potent in HMMA 20.4× more potent in HMMA 38.9× more potent in HMMA
Zavegepant Figure 1 of [23]	pA_2_: 9.92 ± 0.24	pK_b_ 10 nM: 10.02 ± 0.07	1.3× more potent in HMMA

## Data Availability

Data is contained within the article.
